# Notes and Notices

**Published:** 1887-12

**Authors:** 


### NOTES AND NOTICES.

MARRIED.—Wilson—Wallace: Dr. Chas. G. Wilson, of Clarksville, Tenn., to Miss Carrie K. Wallace, by Rev. J. T. Hargrave, September 29th, 1887.

ANTIPATHIES.—Cassell's magazine, *The Quiver*, contains a curious article on the subject of antipathies. Peter the Great could not bear the sight of running water, and Henry III of France fainted at the sight of a cat. The unhappy Princess de Lamballe had a horror of violets, and Vincent, the painter, swooned from the smell of roses.

EARLY USE OF THE MAGNET IN SURGERY.—In the *Philological Magazine* for August, 1808, there is an interesting account of a successful operation performed by the aid of a magnet, in which a small piece of iron, which had embedded itself in the eye of a blacksmith, was extracted after resisting the efforts of the surgeons for no less than five months. So far as we are aware, this is the earliest record of the use of the magnet in surgery.—*Electrician.*

OLD STYLE.—"Well, Sambo, are you over your rheumatism?" "Tank you kindly, sah, I's convalescing fast, but I don't get no better."

A RARA AVIS.—We do not know at present of a single absolute follower of Hahnemann! A small minority, who profess much, go far beyond him in their dilution of drugs, and vary from his precepts in many other respects, while the great majority of those who style themselves homœopathists do not even pretend to follow his methods! If there was any one mode of treatment which Hahneman condemned more than another, it was the use of local applications in affections of the skin, and still we hear of professed homœopathists recommending this anti-Hahnemannian treatment in high places—yes, even in the very sanctuary of homœopathic pretension!

There are some methods of Hahnemann which will stand the test of all time, and suffering humanity cannot be too thankful for their having been promulgated!—*N. Y. Medical Times.*

WORMS IN EGGS.—Professor Liebe adduces reliable data in answer to the question whether living worms are to be found in hen's eggs. A short time previously his sister had found a round, thread-like worm, the length of a little finger, in the white of an egg. It moved itself in a very lively manner. She at once took the white of the egg to a druggist, who put the worm in alcohol. Professor Mobius, of Kiel, decided that the specimen was an example of the thread-worm of fowls—*Heteratis inflexa*—often found in the small intestines of the domestic hen. Only a few instances of the existence of the same in the white of the egg have been recorded.—*Allgemeine Medicinische Central-Zeitung.*

THE LADIES' AID ASSOCIATION of the National Homœopathic Hospital of Washington, D. C., have issued an urgent appeal to every homœopathic physician in the land in aid of this institution. In their pamphlet they present four ways in which doctors at a distance may assist them: First, by the doctor's suggesting a suitable person in his own neighborhood to act as Vice-President, and the names of two energetic women who will aid the Society in the capacity of a State Committee. Secondly, by each doctor becoming an annual member of the Association on payment of one dollar. Thirdly, by persuading friends and patients to become members and make endowments. Fourthly, by presenting the subject at meetings of State Medical Conventions and enlisting sympathy. Copies of the circular may be had of Mrs. Lida Nordhoff, 1731 K Street, N. W., Washington, D. C.

THE SOUTHERN HOMŒOPATHIC MEDICAL ASSOCIATION will hold its fourth annual meeting in New Orleans December 14th, 15th, and 16th, 1887.

NEW HOMŒOPATHIC DISPENSARY.—The homœopathic physicians of Sedalia, Mo., have established a free dispensary. Dr. C. S. Durant will be in charge, assisted by his brethren when help is needed. May the dispensary soon have all it can attend to; there is no better way of showing what Homœopathy can do than by dispensary work.

GIVE NATURE A CHANCE.—Secretary of State Bayard, in welcoming the members of the recent International Medical Congress, said, among other things: "Forgive me if, as one of the great army of patients, I humbly petition the profession that in your deliberations Nature may be allowed a hearing when remedies are proposed; that her *vis medicatrix* may not be omitted in computing the forces of cure, and that science may be restricted as often as possible to sounding the alarm for Nature to hasten, as she surely will, if permitted, to the defense of the point assailed."

THE INDEX TO VOLUME SEVENTH.—We call the attention of our readers to the very thorough index accompanying this number. We believe it to be the most complete of any medical journal. It will be seen that not only is it possible to find every article under its most important word, but there is also a complete set of references to every remedy wherever mentioned. One of the most important characters of every book must be a copious index. Nothing more quickly attracts the criticism of the literary man. In the present issue we believe we have that highly desirable addition.

A SUBSCRIBER'S OPINION.—No true homœopath should fail to subscribe to THE HOMŒOPATHIC PHYSICIAN. For to sustain it is to hold up the homœopathic flag in its true colors. Long may it wave: and may its every flutter in the mongrel and "regular" breeze signify victory for the true healing art.

J. C. HOLLOWAY, M. D.

